# Exploring the evolutionary process of alkannin/shikonin *O*-acyltransferases by a reliable *Lithospermum erythrorhizon* genome

**DOI:** 10.1093/dnares/dsab015

**Published:** 2021-08-23

**Authors:** Chengyi Tang

**Affiliations:** School of the Environment, Nanjing University, Nanjing, China

**Keywords:** *Lithospermum erythrorhizon* genome, alkannin/shikonin *O*-acyltransferases, gene duplication, positive selection

## Abstract

Increasing genome data are coming out. Genome size estimation plays an essential role in guiding genome assembly. Several months ago, other researchers were the first to publish a draft genome of the red gromwell (i.e. *Lithospermum erythrorhizon*). However, we considered that the genome size they estimated and assembled was incorrect. This study meticulously estimated the *L. erythrorhizon* genome size to should be ∼708.74 Mb and further provided a reliable genome version (size ≈ 693.34 Mb; contig_N50_ length ≈ 238.08 Kb) to support our objection. Furthermore, according to our genome, we identified a gene family of the alkannin/shikonin *O*-acyltransferases (i.e. AAT/SAT) that catalysed enantiomer-specific acylations in the alkannin/shikonin biosynthesis (a characteristic metabolic pathway in *L. erythrorhizon*’s roots) and further explored its evolutionary process. The results indicated that the existing AAT/SAT were not generated from only one round of gene duplication but three rounds; after different rounds of gene duplication, the existing AAT/SAT and their recent ancestors were under positive selection at different amino acid sites. These suggested that a combined power from gene duplication plus positive selection plausibly propelled AAT/SAT’s functional differentiation in evolution.

## 1. Introduction

**Figure 1:**
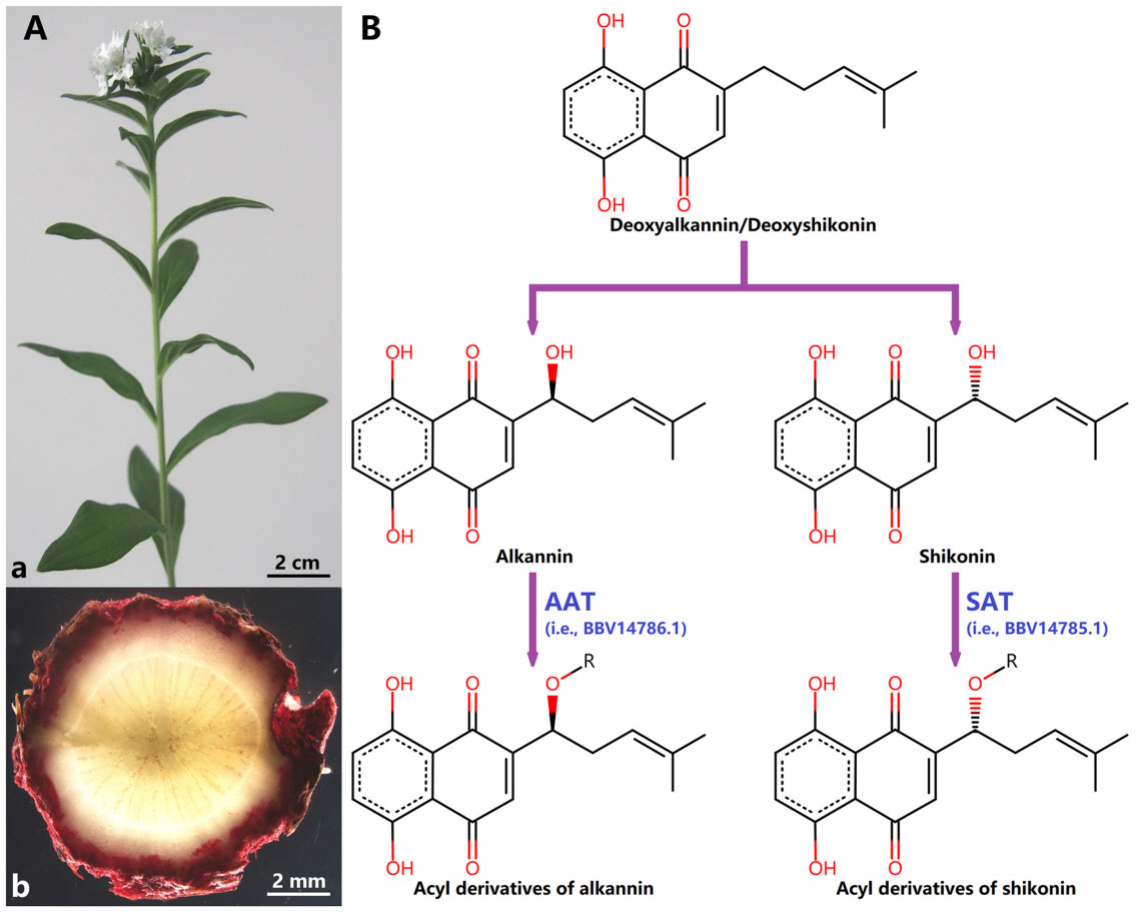
*Lithospermum erythrorhizon* and alkannin/shikonin’s acylation reactions. (A) An individual *L. erythrorhizon* in our greenhouse. a. Leaves, stems, and flowers; b. Roots’ cross-section. (B) Alkannin/shikonin’s enantiomer-specific *O*-acylations. AAT: alkannin *O*-acyltransferase (NCBI ID: BBV14786.1); SAT: shikonin *O*-acyltransferase (NCBI ID: BBV14785.1).

Red gromwell (Fig. 1A), i.e. *Lithospermum erythrorhizon* Siebold & Zucc., is a traditional Chinese medicine plant [former name: *L. officinale* var. *erythrorhizon* (Siebold & Zucc.) Maxim.^1^; No. of chromosomes: all records 2n = 28^2^]. In the past, *L. erythrorhizon* was recognized as a variant of *L. officinale* L. (former name: *L. officinale* var. *stewartii* Kazmi^1^; No. of chromosomes: most records 2n = 28^2^), although they are now separated species. Besides, based on current molecular evidence, *L. erythrorhizon* is still determined as the closest species of *L. officinale.*^3^

Lately, Auber et al.^4^ published a hybrid assembled genome of *L. erythrorhizon* (estimated genome size ≈ 369.34 Mb; assembled genome size ≈ 367.41 Mb) using our short Illumina data (NCBI ID: SRX2882373; Supplementary Table S1) plus their long ONT data (NCBI IDs: SRX7432848–SRX7432852; Supplementary Table S1). However, Pustahija et al.^5^ reported that *L. officinale’*s genome size was ∼743 Mb (1C ≈ 0.76 pg), significantly greater than the *L. erythrorhizon’*s genome size estimated and assembled by Auber et al.^4^ Since the chromosome numbers between *L. erythrorhizon and L. officinale* are almost identical,^2^ we consider that this significant difference is not due to polyploidization but to Auber et al.’s misestimation and misassembly.^4^ Therefore, in this study, we carried out a rigorous genome size estimation and further provided a new version of the *L. erythrorhizon* genome to support our objection.

Alkannin/shikonin and their acyl derivatives are main secondary-metabolites in *L. erythrorhizon’*s root periderm (Fig. 1A).^4^^,^^6^ Recently, Oshikiri et al.^7^ verified that two enzymes (NCBI IDs: BBV14785.1 and BBV14786.1) were enantiomer-specific alkannin/shikonin *O*-acyltransferases (i.e. AAT/SAT; Fig. 1B) in *L. erythrorhizon*. However, AAT/SAT family members in *L. erythrorhizon* and their evolutionary process were still indistinct. Therefore, we detailedly identified AAT/SAT-like superfamily members in the genomes of *L. erythrorhizon* plus other nine representative species (Supplementary Table S2),^8–16^ and further conducted a series of analyses to illuminate AAT/SAT’s evolutionary process.

## 2. Materials and methods

### 2.1. Plant materials

The seeds of *L. erythrorhizon* were purchased from ShiJie Seed Breeding Company (https://cfsjzy.1688.com/), located in Chifeng, Inner Mongolia Autonomous Region, China. The healthy seeds were germinated in several pots and then cultured in a greenhouse. Young leaves from flowering individuals were applied for genome sequencing.

### 2.2. Genome sequencing

Genomic DNA was extracted using a Magnetic Plant Genomic DNA Kit (Cat. no: 4992407; Tiangen, China). After quality control, a paired-end library (insert size ∼170 bp) was constructed using a TIANSeq Fast DNA Library Kit (Cat. no: 4992261; Tiangen, China) and then was sequenced by an Illumina HiSeq 2000 sequenator (Illumina, USA). Subsequently, a SMRTbell library (∼20 Kb) was constructed using a SMRTbell Express Template Prep Kit (PN: 100-938-900; PacBio, USA) and then was sequenced by a PacBio Sequel sequenator (PacBio, USA).

### 2.3. Data processing

Trimmomatic v0.36^17^ and FastUniq v1.1^18^ filtered Illumina raw data to remove adapters, low-quality reads, poly-N reads, and PCR-duplicated reads. SMRT Link v6.0 (https://www.pacb.com/support/software-downloads) filtered PacBio raw data to remove adapters and too-short reads (i.e. length < 1 Kb). Furthermore, NanoFilt v2.5.0^19^ filtered ONT raw data that Auber et al. published (NCBI IDs: SRX7432848–SRX7432852; Supplementary Table S1)^4^ to remove too-short reads (i.e. length < 1 Kb) and low-quality reads (i.e. RQ < 7.0).

### 2.4. Genome size estimation

Illumina clean data were applied to estimate genome sizes: (i) kmers were counted, and then were exported to histogram files using Jellyfish v2.2.10^20^ (key parameter: jellyfish histo-h Max_count); (ii) GenomeScope v1.0,^21^ GenomeScope v2.0,^22^ and GCE v1.0.2^23^ with the corresponding key parameters (Supplementary Table S3) were applied to calculate genome sizes, respectively. Furthermore, the chloroplast reads were removed in total Illumina clean data via BWA v0.7.17^24^ (key parameter: bwa mem) and SAMtools v1.10^25^ (key parameter: samtools view -bF 4) based on three Lithospermeae chloroplast genomes downloaded from NCBI (NCBI IDs: MT975394.1, MT975393.1, and NC_049569.1; Supplementary Table S4).^26^^,^^27^ Subsequently, the cpclean data (i.e. chloroplast-filtered) also were used to estimate genome sizes according to the identical steps mentioned above.

### 2.5. Genome assembly and annotation

PacBio and ONT clean data were first corrected via NextDenovo v2.3.1 (key parameter: read_cutoff = 1,000; seed_cutoff = 10,000) (https://github.com/Nextomics/NextDenovo), separately. The total corrected data were then applied for genome assembly using NextDenovo v2.3.1 (key parameter: nextgraph_options = –a 1), SmartDenovo v1.0.0^28^ (key parameter: –J 5000; –k 16), Flye v2.8.1^29^ (key parameter: –i 1), and Wtdbg v2.5^30^ (key parameter: –L 5000; –k 15; –p 0; –S 2), independently. Finally, the NextDenovo-assembled version was further polished three rounds via Pilon v1.23^31^ based on Illumina clean data. In addition, BUSCO v2.0.1^32^ was applied to evaluate genome completeness.

Repetitive sequences were identified via RepeatMasker v4.1.1 (http://www.repeatmasker.org) based on a combined database including curated Dfam v3.2,^33^ RepBase (RepeatMasker Edition-20181026),^34^ plus a custom *L. erythrorhizon* library constructed via RepeatModeler v2.0.1 (key parameter: -LTRStruct) (http://www.repeatmasker.org/RepeatModeler). Subsequently, protein-coding genes were predicted as the following process: (i) repetitive sequences were masked first; (ii) AUGUSTUS v3.3.3,^35^ GlimmerHMM v3.0.4,^36^ and SNAP^37^ were used for *ab initio* prediction; (iii) GeMoMa v1.6.4^38^ was applied for homology prediction based on four published genome data (Supplementary Table S2)^8^^,^^10^^,^^12^^,^^16^; (iv) PASA v2.4.1^39^ and TransDecoder v5.5.0 (https://github.com/TransDecoder/TransDecoder) were used to identify transcripts based on transcriptome data that we published previously (NCBI IDs: SRX3978407–SRX3978409; Supplementary Table S1)^6^; (v) total results were finally integrated into a union set without overlap using EVidenceModeler v1.1.1.^40^

### 2.6. Identification of AAT/SAT-like superfamily

The identification process was as follows: (i) with AAT/SAT’s amino acid sequences (i.e. BBV14785.1 and BBV14786.1) as the queries and 10 genomes (i.e. *L. erythrorhizon* plus nine representative species; Supplementary Table S2) as a database, similarity searches were severally performed using DIAMOND v2.0.5^41^ (key parameter: -f 6 –more-sensitive -e 1e-5 -k 1000); (ii) the redundant sequences were first removed in the results; (iii) then, unusual sequences (i.e. containing abnormal bases, lacking initiation codon and/or termination codon) were filtered out; (iv) batch CD-Search^42^ was used to further identify protein domains (key parameter: e-value: 1e-5; database: Pfam v32.0^43^); since AAT/SAT contained only one complete domain (i.e. PF02458|Transferase) as their characteristic structure (Supplementary Table S11),^44–46^ the sequences containing redundant domains and/or incomplete PF02458 domain are deleted; (v) finally, MEME v5.2.0^47^ was applied to search and identify protein motifs (key parameter: –mod oops –nmotifs 20 –minw 5 –maxw 100; e-value for search: 1e-1000; e-value for identification: 1e-5); since AAT/SAT contained two characteristic motifs (i.e. HXXXD and DFGWG; the DFGWG motif is not absolutely conservative; Supplementary Table S12),^44–46^ the sequences containing both these two motifs were retained as AAT/SAT-like superfamily members. Furthermore, according to the Swiss-Prot database, we applied DIAMOND v2.0.5^41^ (key parameter: -f 6 -e 1e-100 –id 99 -k 3) to confirm which members had been functionally verified by previous studies.

### 2.7. Phylogenetic analysis

The amino acid sequences of the identified AAT/SAT-like superfamily members were aligned via MUSCLE v3.8.31.^48^ Subsequently, the preliminary alignment was trimmed using trimAl v1.4.1^49^ (key parameter: –gt 0.50). The trimmed alignment was used to construct a phylogenetic tree via IQ-TREE v2.0.3^50^ according to the maximum likelihood (i.e., ML) method (best-fit model: VT + F + R10; key parameter: –seqtype AA -m MFP –alrt 1000 -B 1000). Furthermore, we distinguished the AAT/SAT-like family according to the tree structure.

Based on the codon model, the *L. erythrorhizon’*s AAT/SAT-like family members’ nucleotide sequences were aligned via PRANK v170427^51^ (key parameter: -F -codon). Then, the preliminary alignment was trimmed by trimAl v1.4.1^49^ (key parameter: –gt 0.50). The trimmed alignment was transformed back to amino acid sequences, and this amino acid alignment was used to construct a phylogenetic tree via MEGA-X^52^ based on the ML method (best-fit model: JTT + G4; bootstrap replications: 1,000). Besides, this tree and its trimmed codon alignment were used for the following selection pressure analysis.

### 2.8. Ks calculation

Total 12 *L. erythrorhizon’*s AAT/SAT-like family members combined to produce 66 gene pairs C122. Each gene pair was aligned via MUSCLE v3.8.31^48^ based on the corresponding amino acid sequences, and each alignment was transformed back to nucleotide sequences. Ks values for each gene pair were calculated via KaKs_Calculator v2.0^53^ (key parameter: –m NG).

### 2.9. Gene duplication identification

Our *L. erythrorhizon* gene set was applied for all-vs.-all similarity searches via DIAMOND v2.0.5^41^ (key parameter: -f 6 –more-sensitive -e 1e-30 -k 6). The results plus the corresponding gff file of the gene set were further input into the ‘duplicate_gene_classifier’ module in MCScanX^54^ to identify duplication types for each gene (priority: WGD/Segmental > Tandem > Proximal > Dispersed > Singleton).

### 2.10. Selection pressure analysis

According to the branch-site models, the CodeML module in PAML v4.9j^55^ was used to detect positive sites on foreground branches: (i) first, a target foreground branch was labelled in the corresponding tree; (ii) an alternative model (i.e. Model A) was set to that sites were under positive selection on the labelled foreground branch (key parameter: model = 2, NSsites = 2, fix_omega = 0, omega = 1.5); (iii) a null model (i.e. Model A null) was then set to that sites were under neutral selection on the labelled foreground branch (key parameter: model = 2, NSsites = 2, fix_omega = 1, omega = 1); (iv) the likelihood ratio test (i.e. LRT)^56^ was then applied to determine which model was accepted [threshold: when *P *<* *0.05, the alternative model (i.e. Model A) was accepted], (v) furthermore, the bayes empirical bayes test (i.e., BEB)^57^ was used to determine which site was under positive selection (threshold: when *posterior probabilities* > 0.90, that site probably was under positive selection).

## 3. Results and discussion

### 3.1. *Lithospermum Erythrorhizon* genome

Based on our Illumina data [NCBI ID: SRX2882373 (SRR5644206); Supplementary Table S1], Auber et al.^4^ estimated *L. erythrorhizon’*s genome size to be ∼369.34 Mb using GenomeScope v1.0^21^ with default parameters (i.e. parameter ‘Kmer length’ = 21 and parameter ‘Max kmer coverage’ = 1e + 03). We repeated their calculation and obtained an identical result (Supplementary Table S3 and Fig. S1). The original intention of setting parameter ‘Max kmer coverage’ = 1e + 03 was to avoid interference from high-frequency non-nuclear reads (e.g. organelle reads and contamination reads).^21^ However, the practice had proven that this obsolete default parameter (i.e. ‘Max kmer coverage’ = 1e + 03) was improper (https://github.com/schatzlab/genomescope/issues/22; https://github.com/schatzlab/genomescope/issues/28). Thus, software developers suggested this parameter to be set to 1e + 06 (https://github.com/schatzlab/genomescope/issues/30), and further changed this default from 1e + 03 to all (i.e. ‘Max kmer coverage’ = –1) in the GenomeScope latest version (i.e. v2.0).^22^

**Figure 2:**
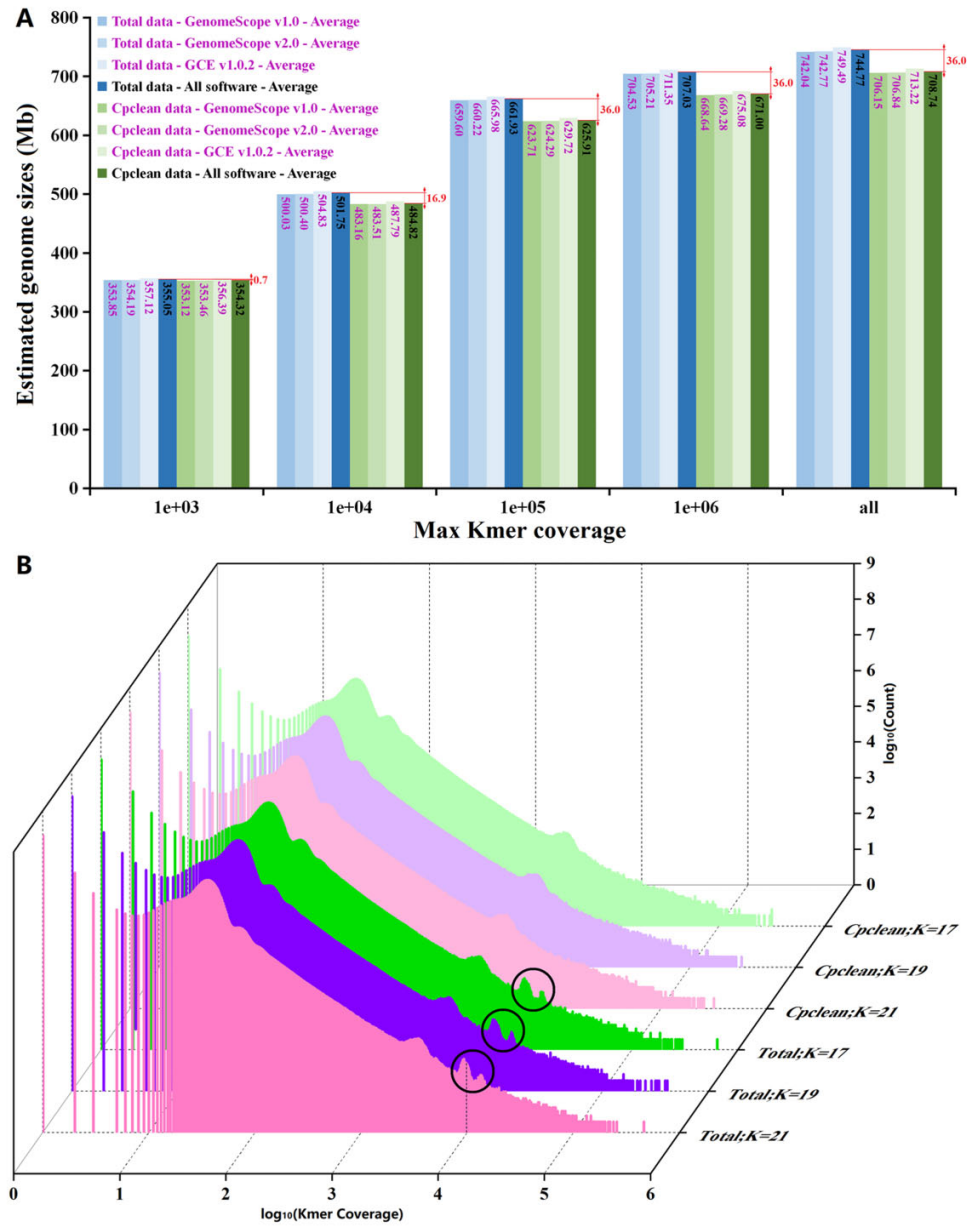
*Lithospermum erythrorhizon’*s estimated genome sizes and kmer frequency histogram. Total: total clean Illumina data; Cpclean: chloroplast-filtered data. (A) Estimated genome sizes at five thresholds of parameter ‘Max kmer coverage’ (i.e. 1e + 03, 1e + 04, 1e + 05, 1e + 06, and all) via using GenomeScope v1.0, GenomeScope v2.0 and GCE v1.0.2. (B) Kmer frequency histograms at three thresholds of parameter ‘Kmer length’ (i.e. K = 17, 19, and 21). Black circles: the peaks of the chloroplast reads.

For Spermatophyta, high-frequency non-nuclear reads primarily come from chloroplast because current materials used for genome sequencing are generally green leaves rather than etiolated leaves. Accordingly, through applying GenomeScope v1.0,^21^ GenomeScope v2.0,^22^ and GCE v1.0.2,^23^ we calculated the *L. erythrorhizon’*s genome size at five thresholds of parameter ‘Max kmer coverage’ (i.e. 1e + 03, 1e + 04, 1e + 05, 1e + 06, and all) with three levels of parameter ‘Kmer length’ (i.e. 17, 19, and 21), based on total Illumina data and corresponding chloroplast-filtered data (i.e. cpclean data; Supplementary Table S4). The results (Fig. 2A and Supplementary Table S3) showed that: (i) different software (or versions) and parameter ‘Kmer length’ had little effect on genome size estimation when parameter ‘Max kmer coverage’ was fixed; (ii) the estimated genome sizes continued to increase as ‘Max kmer coverage’ became large; (iii) when ‘Max kmer coverage’ ≥ 1e + 04, the genome sizes estimated by total data were significantly greater than that estimated by cpclean data; but, the corresponding differences remained almost constant (∼36.0 Mb; Fig. 2A) when ‘Max kmer coverage’ ≥ 1e + 05; these suggested that chloroplast reads significantly skewed the estimated genome size, and these reads mainly concentrated at around ‘Kmer coverage’ ≈ 1e + 04, consistent with the kmer distribution (Fig. 2B) and previous study^21^; (iv) coincidently, the genome size (∼707.03 Mb) estimated by total data at ‘Max kmer coverage’ = 1e + 06 was approximately equal to the size (∼708.74 Mb) estimated by cpclean data at ‘Max kmer coverage’ = all, due to the increased size caused by chloroplast reads exactly offset the decreased size caused by a lack of high-kmer reads (i.e. the reads at ‘Max kmer coverage’ > 1e + 6); this probably was why developers first suggested parameter ‘Max kmer coverage’ to be set to 1e + 06 and further changed it to all; in other words, to make the calculation more accurate, parameter ‘Max kmer coverage’ was recommended to be set to all when high-frequency non-nuclear reads can be filtered out, whereas this parameter was suggested to be set to 1e + 06 as an empirical value when high-frequency non-nuclear reads cannot be filtered out due to a lack of reference databases (e.g. a chloroplast genome).^22^ Therefore, we believed that the actual *L. erythrorhizon’*s genome size should be ∼708.74 Mb, which approached the *L. officinale’*s genome size (∼743 Mb) as Pustahija et al. reported^5^ rather than ∼369.34 Mb as Auber et al. estimated.^4^

Additionally, we noticed Auber et al.’s descriptions about our Illumina data [NCBI ID: SRX2882373 (SRR5644206); https://www.ncbi.nlm.nih.gov/sra/SRX2882373; Supplementary Table S1]^4^ were incorrect. Auber et al. wrote in their article:

To create a reference genome, we combined *L. erythrorhizon* ONT genomic DNA (gDNA) reads generated in-house from Siebold & Zucc. Plants with publicly available Illumina gDNA reads sequenced by Nanjing University in 2018 from an unknown accession (SRR5644206). The Illumina data consisted of ∼21.7 Gb Illumina HiSeq paired-end short reads (150 bp) with an estimated heterozygosity of 0.39% and projected genome size of 369.34 Mb.^4^

In fact, these Illumina data were not an unknown accession. We can know that the submitter was Dr. Chengyi Tang by inquiring about the corresponding BioSample ID (NCBI ID: SAMN06972300; https://www.ncbi.nlm.nih.gov/biosample/SAMN06972300). And, these data should contain a total of 173,693,157 × 2 paired-end reads. The corresponding total length should be ∼34.7 Gb (Gigabases) (Supplementary Table S1). The ‘∼21.7 Gb (Gigabytes)’ Auber et al. wrote^4^ was just a computer file size. The reads length should be 100 bp rather than 150 bp (i.e. SpotLen = 200 = 100 × 2). All these corresponding statistical information had already been published in NCBI on 2018-04-15 (https://www.ncbi.nlm.nih.gov/Traces/study/?acc=SRX2882373&o=acc_s%3Aa). In view of Auber et al.’s thoughtless attitude for reference data, we have no faith in Auber et al.’s ability to acquire an actual *L. erythrorhizon* genome size via our data.

Furthermore, we assembled the *L. erythrorhizon* genome. The total long reads used for assembling were 31.5 Gb (Supplementary Table S1). The sizes of the preliminary assembled genomes were 680.91–745.64 Mb (Supplementary Table S5). Again, this proved that the actual genome size should be ∼708.74 Mb as estimated above rather than ∼369.34 Mb as Auber et al. reported.^4^ If the estimated genome size was ∼369.34 Mb, the data coverage could reach up to 85.29× (i.e. 31.5 Gb/369.34 Mb ≈ 85.29); with such sufficient coverage, it is impossible that the estimated size (∼369.34 Mb) was significantly lesser than the assembled size (680.91∼745.64 Mb). The NextDenovo-assembled version was then selected for an error correction because its size and continuity were better than others (Supplementary Table S5). The final genome size was ∼693.34 Mb, and the contig_N50_ length was ∼238.08 Kb (Supplementary Table S5). BUSCO evaluation showed that ∼88.68% of complete BUSCOs from Embryophyta.odb9^32^ could map to our genome (i.e. 1,277/1,440; Supplementary Table S6), indicating that our genome completeness was acceptable and better than Auber et al.’s (their mapped ratio was only ∼79.31%,^4^ i.e. 1,141/1,440). Subsequently, we predicted that our genome contained ∼480.93 Mb (∼69.36%) repetitive sequences in which tandem repeats were ∼4.76 Mb and interspersed repeats were ∼472.45 Mb (Supplementary Table S7); and, our genome also contained 35,932 protein-coding genes, in which 28,995 genes (∼80.69%) were supported by transcriptome data (Supplementary Table S8 and Fig. S2).

### 3.2. AAT/SAT-like family

**Figure 3:**
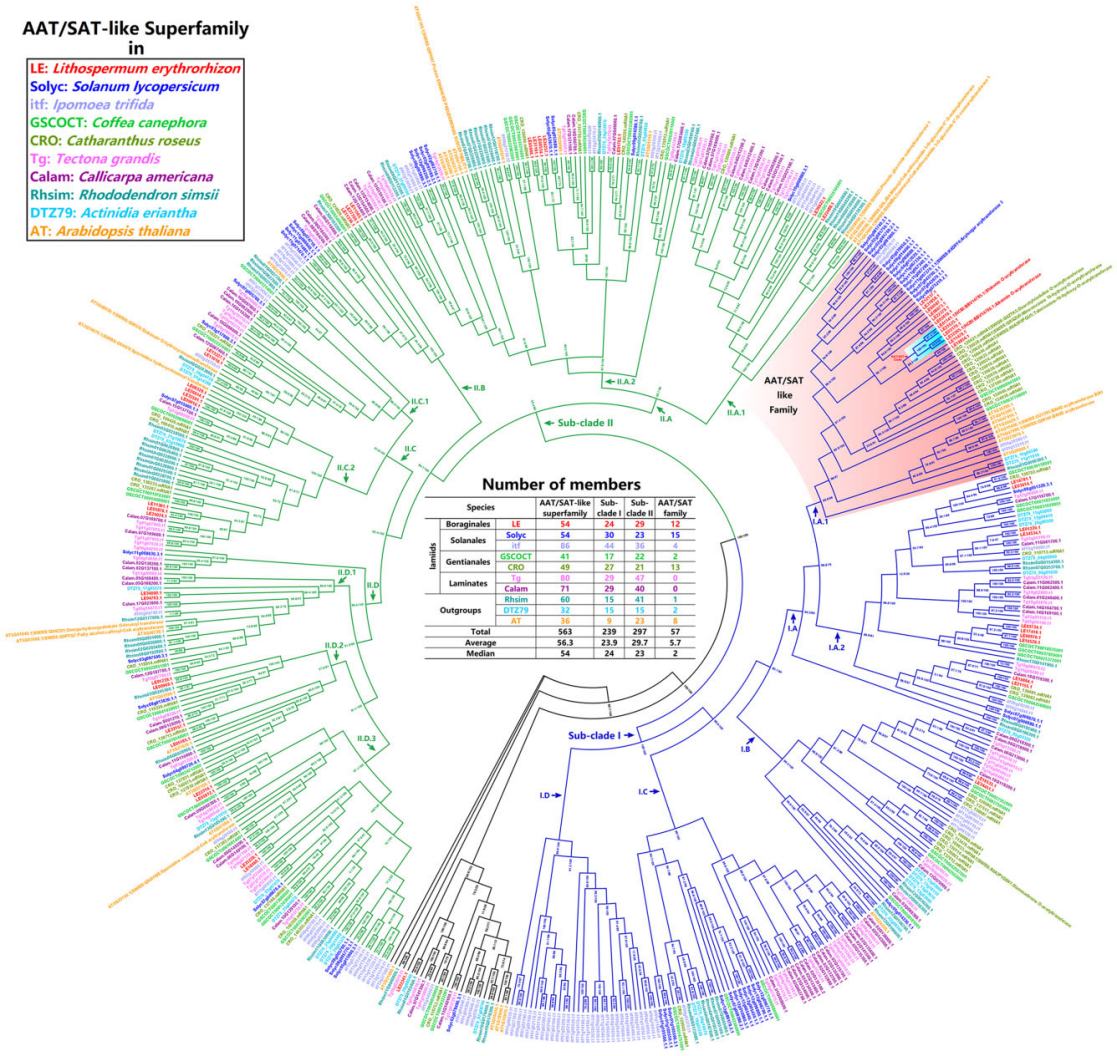
An ML phylogenetic tree of the AAT/SAT-like superfamily across 10 representative species (i.e. seven lamiids species plus three outgroup species; Supplementary Table S1). */* is SH-aLRT support/ultrafast bootstrap support. Two main sub-clades: Sub-clade I (blue branches) and Sub-clade II (green branches). The Sub-clade I contains five sub-categories, i.e. I.A.1∼2, I.B, I.C, and I.D; the Sub-clade II contains eight sub-categories, i.e. II.A.1∼2, II.B, II.C.1∼2, and II.D.1∼3. The sub-category I.A.1 is named as ‘AAT/SAT-like family’ in this study.

*Lithospermum* *erythrorhizon* belongs to Boraginales; and, Boraginales, together with three other orders (i.e. Solanales, Gentianales, and Laminates), are the four core groups in the lamiids clade.^6^^,^^58^ Therefore, through sequence similarity search, we identified 1,233 AAT/SAT-like genes (Supplementary Table S9) in *L. erythrorhizon*, six other lamiids species (i.e. two Solanales species: *Solanum lycopersicum*, *Ipomoea trifida*; two Gentianales species: *Coffea canephora*, *Catharanthus roseus*; two Lamiales species: *Tectona grandis*, *Callicarpa americana*), and three outgroup species (i.e. *Rhododendron simsii*, *Actinidia eriantha*, and *Arabidopsis thaliana*) (Supplementary Table S2). As expected, we found that the AAT’s equivalent was LE32265.1, and the SAT’s equivalent was LE01141.1 (Supplementary Table S9). After removing redundant sequences and unusual encoding sequences, a total of 674 genes were retained (Supplementary Table S10). Since AAT/SAT contained one characteristic domain (i.e. PF02458|Transferase) and two characteristic motifs (i.e. HXXXD and DFGWG) (Supplementary Tables S11 and S12),^44–46^ the sequences containing abnormal domains and motifs were further filtered. Finally, a total of 563 genes (Fig. 3 and Supplementary Table S12) were retained as AAT/SAT-like superfamily members, in which at least 18 members had been verified by previous studies [i.e. 2 (i.e. AAT/SAT) + 16 from the Swiss-Prot database (Supplementary Table S13)]. According to the above structural and functional information, the AAT/SAT-like superfamily should be the BAHD superfamily (i.e. benzylalcohol *O*-acetyltransferase, anthocyanin *O*-hydroxycinnamoyltransferase, *N*-hydroxycinnamoyl anthranilate benzoyltransferase, and deacetylvindoline *O*-acetyltransferase superfamily), which catalysed various acylation reactions in plant metabolism (e.g. lignins, anthocyanins, terpenoids, and various esters).^44–46^

To further classify the AAT/SAT-like superfamily, we constructed a phylogenetic tree. The results (Fig. 3) showed that: (i) this superfamily was roughly divided into three sections, i.e. Sub-clade I, Sub-clade II, and some oddments; furthermore, the Sub-clade I was roughly divided into four broad categories and five sub-categories (i.e. I.A.1∼2, I.B, I.C, and I.D), and the Sub-clade II was roughly divided into four broad categories and eight sub-categories (i.e. II.A.1∼2, II.B, II.C.1∼2, and II.D.1∼3); (ii) AAT/SAT belonged to the sub-category I.A.1; thus, we named this sub-category as ‘AAT/SAT-like family’ in this study; in addition, this AAT/SAT-like family (i.e. the sub-category I.A.1) contained a total of eight verified members, in which CRO_120021.mRNA1 (i.e. Swiss-Prot ID: Q9ZTK5|deacetylvindoline *O*-acetyltransferase from *C. roseus*; Supplementary Table S13) was used to name the BAHD superfamily in previous studies^44–46^; (iii) although *S. lycopersicum and I. trifida* belonged to Solanales, the numbers of the AAT/SAT-like family members they each contained were significantly different (i.e. *S. lycopersicum*: 15 vs. *I. trifida*: 4; Fig. 3), and a similar numerical difference was also found in two Gentianales species (i.e. *C. canephora*: 2 vs. *C. roseus*: 13; Fig. 3); in addition, two Lamiales species (i.e. *T. grandis and C. americana*) did not contain any members in this AAT/SAT-like family, although they owned abundant members in the Sub-clade I and the whole superfamily (Fig. 3); therefore, all these indicated that the number of AAT/SAT-like family members significantly expanded or contracted in different species, which might be related to the species-specific properties.

### 3.3. AAT/SAT’s evolutionary process

In the AAT/SAT-like family (i.e. the sub-category I.A.1), AAT/SAT (i.e. LE32265.1 and LE01141.1) plus two other members (i.e. LE03170.1 and LE25525.1) seem to converge into a common clade (Fig. 3). Therefore, we named this clade as ‘AAT/SAT clade’ in this study. These four members should be real gene loci because the evidence collectively supports them on three fronts (i.e. *Ab initio* + Homology + Transcriptome; Supplementary Table S8). Based on the tree reconstructed only using 12 *L. erythrorhizon* members (Supplementary Fig. S3) and the corresponding Ks values (Supplementary Table S14), we further reconfirmed that these four members should be descended from a recent common ancestor (i.e. a common clade) due to Ks_clade I (i.e.__AAT/SAT clade)_ (≈ 0.46) ≪ Ks_clade I vs. clade II_ (≈ 1.97) and Ks_clade I_ ≪ Ks

_clade I vs. clade III_ (≈ 2.52).

**Figure 4.**
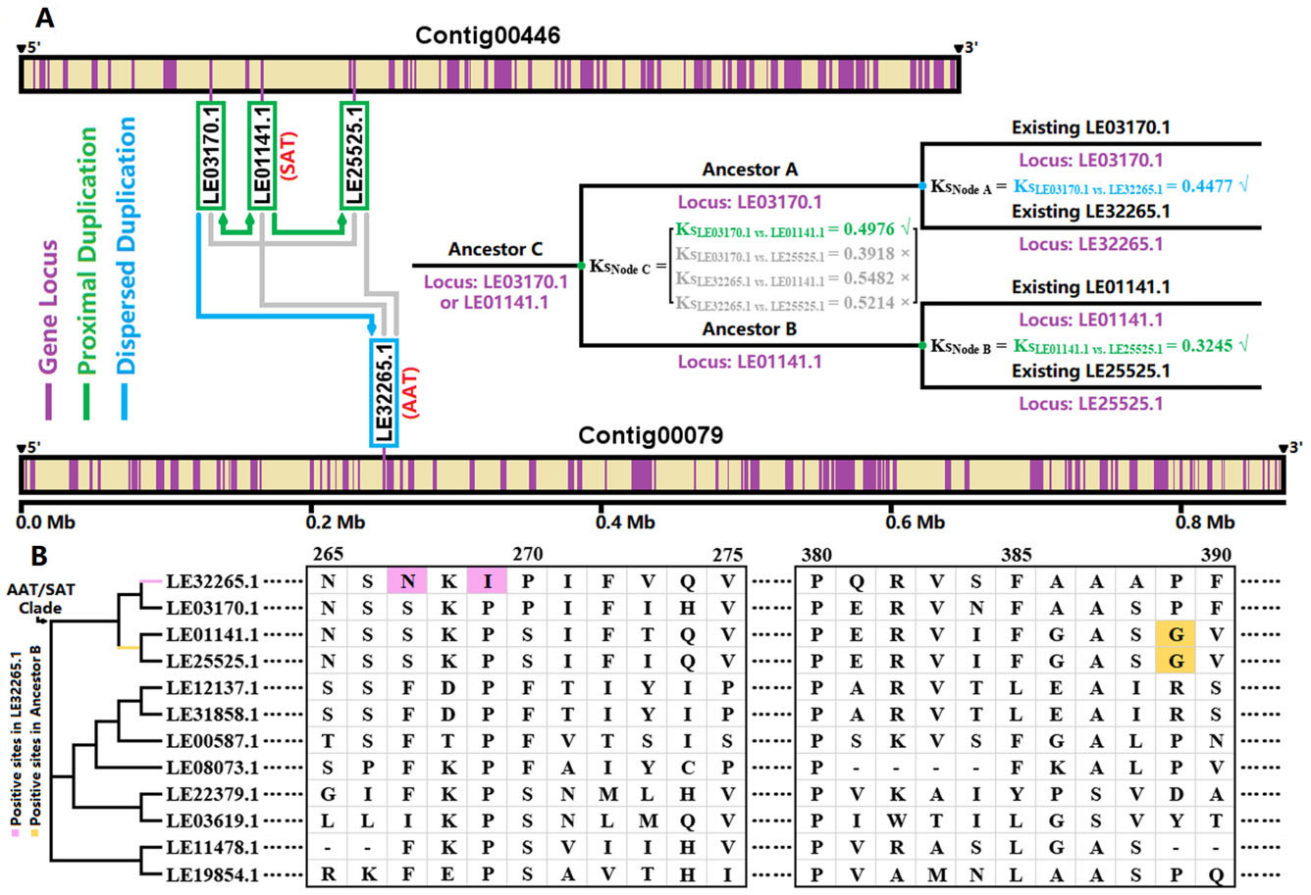
AAT/SAT’s evolutionary process in *L. erythrorhizon*. (A) AAT/SAT and two other members’ locus information and duplication process. ‘√’: these Ks values can be accepted; ‘×’: these Ks values cannot be accepted. The Ks values are based on Supplementary Table S14; and, the duplication types are based on Supplementary Table S15. (B) Potential positive selection sites inside the AAT/SAT clade. The complete amino acid alignment (after trimAl) is exhibited in Supplementary Fig. S4.

Subsequently, we identified duplication types for each gene in the *L. erythrorhizon* genome. The results indicated that three members (i.e. LE01141.1, LE03170.1, and LE25525) came from ‘proximal duplications’, whereas another one (i.e. LE32265.1) came from a ‘dispersed duplication’ (Supplementary Table S15). These were consistent with their loci information in the genome: LE01141.1, LE03170.1, and LE25525.1 were closely located in the Contig00446, whereas LE32265.1 was located in the Contig00079 alone (Fig. 4A). The phylogenetic relationship in the AAT/SAT clade had been confirmed as ((LE32265.1, LE03170.1)_Node A_, (LE01141.1, LE25525.1)_Node B_)_Node C_ (Fig. 3 and Supplementary Fig. S3), and the Ks values between these four members were also known (Supplementary Table S14). Therefore, (i) one round of dispersed duplication should occur in Node A at Ks_Node A_ = Ks_LE03170.1 vs. LE32265.1_ = 0.4477 because only LE32265.1 was identified as ‘dispersed duplication’; (ii) one round of proximal duplication should occur in Node B at Ks_Node B_ = Ks_LE01141.1 vs. LE25525.1_ = 0.3245 because both LE01141.1 and LE25525.1 were identified as ‘proximal duplications’; (iii) and, another round of proximal duplication should occur in Node C at Ks_Node C_ = Ks_LE03170.1 vs. LE01141.1_ = 0.4976 because there was only one round of dispersed duplication in the AAT/SAT clade (thus, Ks_LE32265.1 vs. LE01141.1_ and Ks_LE32265.1 vs. LE25525.1_ were excluded), and Ks_Node C_ must be greater than Ks_Node A_ and Ks_Node B_ (thus, Ks_LE03170.1 vs. LE25525.1_ were excluded) (Fig. 4A). To sum up, we inferred that the AAT/SAT’s evolutionary process probable underwent three rounds of gene duplication (Fig. 4A): (i) first, one round of proximal duplication occurred in Node C at Ks = 0.4976 and made ancestor C produce ancestor A and ancestor B; ancestor A probable located on the existing LE03170.1 locus, and ancestor B probable located on the existing LE01141.1 locus, due to Ks_LE03170.1 vs. LE01141.1_ was assigned to Ks_Node C_; (ii) subsequently, one round of dispersed duplication occurred in Node A at Ks = 0.4477 and made ancestor A produce the existing LE03170.1 and LE32265.1 (i.e. AAT); (iii) finally, another round of proximal duplication occurred in Node B at Ks = 0.3245 and made ancestor B produce the existing LE01141.1 (i.e. SAT) and LE25525.1.

Furthermore, we detected whether positive selection sites existed on each branch inside the AAT/SAT clade. The results showed two potential positive sites (i.e. sites 267 and 269) were on the branch LE32265.1 and one potential positive site (i.e. site 389) was on the branch ancestor B (Fig. 4B and Supplementary Table S16). In other words, (i) after the proximal duplication in Node C, ancestor B was possibly subjected to positive selection, while ancestor A was not; (ii) after the dispersed duplication in Node A, LE32265.1 was possibly subjected to positive selection, while LE03170.1 was not; (iii) after the proximal duplication in Node B, both LE01141.1 and LE25525.1 were not under positive selection. To sum up, the above evidence suggested that gene duplication and positive selection collectively propelled AAT/SAT’s functional differentiation in evolution.

## Supplementary data

Supplementary data are available at *DNARES* online.

## Supplementary Material

dsab015_Supplementary_Data

## Accession numbers

The genome sequencing data of *L. erythrorhizon* were stored in the NCBI, and their accession IDs were SRX2882373 and SRX9956727 (Supplementary Tables S1). The *L. erythrorhizon* genome we assembled was stored in the NCBI (accession ID: JAIEZA000000000), the EMBL (accession ID: CAJVUH010000000) and the CNGBdb (accession ID: CNA0029378), respectively. In addition, the corresponding gene annotation files (i.e. gff/cds/pep) were stored in the CNGBdb, and their shared accession ID was CNA0029378. Other data and genomes downloaded from previous studies were listed in Supplementary Tables S1, S2, and S4.
