# Supplementary material for: Exploring the evolutionary process of alkannin/shikonin O-acyltransferases by a reliable Lithospermum erythrorhizon genome
Source: DNA Res. 2021 Aug 23;28(5):dsab015. doi: 10.1093/dnares/dsab015 (PMC8435551; doi:10.1093/dnares/dsab015)
Supplement: dsab015_Supplementary_Data [file dsab015_supplementary_data.zip › Supplementary Figs S1-S4.pdf]

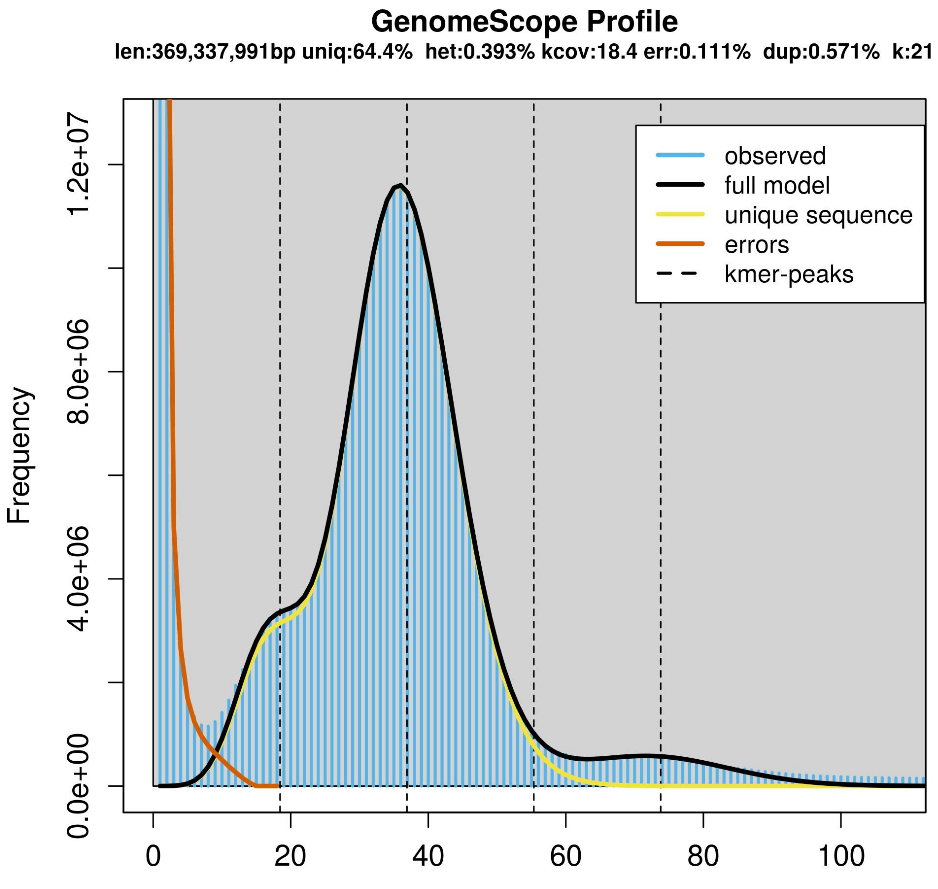

Supplementary Fig. S1. The calculated *L. erythrorhizon* genome size via GenomeScope v1.0, based on parameters "Kmer length = 21" & "Max kmer coverage = 1e+03".

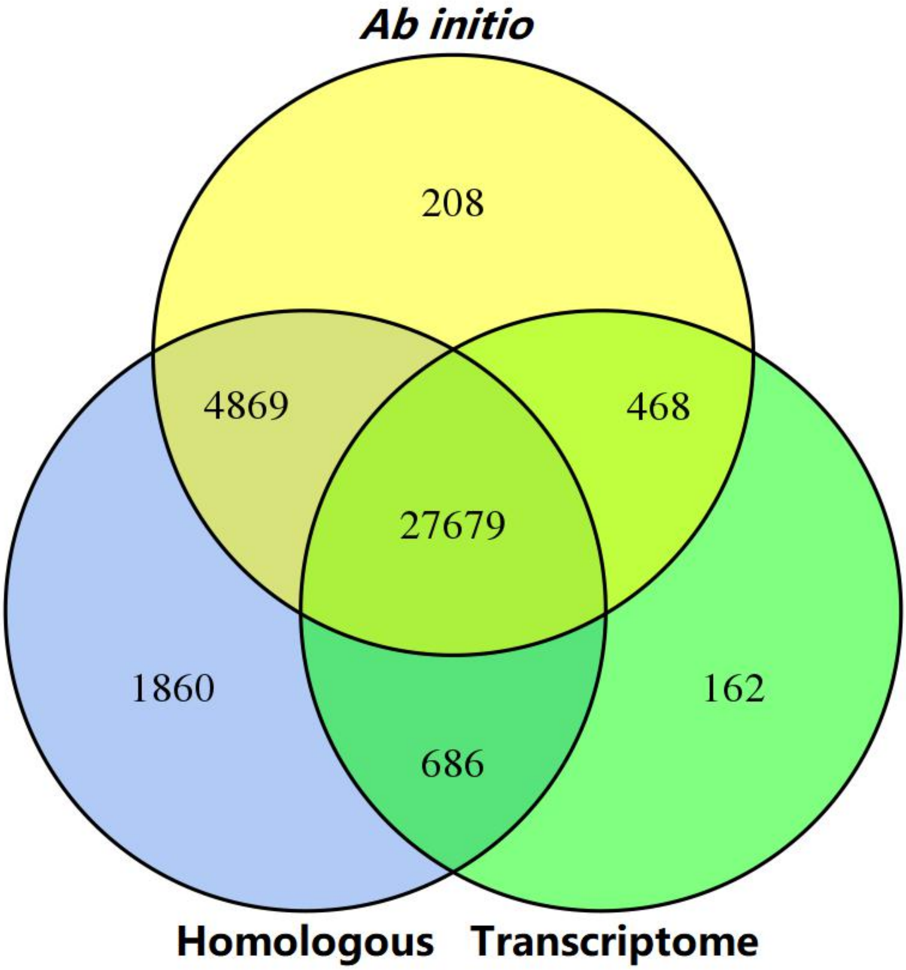

Supplementary Fig. S2. Venn diagram of the supporting evidence of *L. erythrorhizon* gene set.

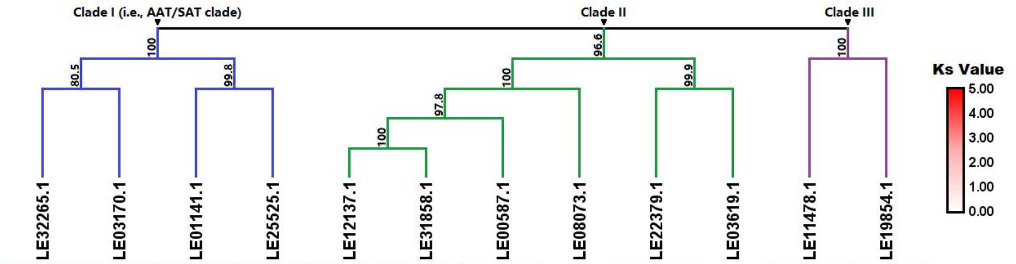

	LE32265.1	LE03170.1	LE01141.1	LE25525.1	LE12137.1	LE31858.1	LE00587.1	LE08073.1	LE22379.1	LE03619.1	LE11478.1	LE19854.1
LE32265.1	0.00	0.45	0.55	0.52	2.27	2.66	1.70	1.58	1.87	1.49	2.74	2.88
LE03170.1	0.45	0.00	0.50	0.39	2.48	2.60	1.60	1.61	2.17	1.67	2.14	2.23
LE01141.1	0.55	0.50	0.00	0.32	2.22	2.13	1.69	1.75	1.67	1.60	2.38	2.19
LE25525.1	0.52	0.39	0.32	0.00	2.48	3.26	1.65	1.58	1.83	1.62	2.54	3.03
LE12137.1	2.27	2.48	2.22	2.48	0.00	0.03	1.80	2.73	2.15	4.15	NaN	NaN
LE31858.1	2.66	2.60	2.13	3.26	0.03	0.00	1.84	2.33	2.05	2.91	NaN	NaN
LE00587.1	1.70	1.60	1.69	1.65	1.80	1.84	0.00	1.78	1.88	1.66	2.99	2.10
LE08073.1	1.58	1.61	1.75	1.58	2.73	2.33	1.78	0.00	2.36	1.70	1.90	1.87
LE22379.1	1.87	2.17	1.67	1.83	2.15	2.05	1.88	2.36	0.00	1.16	2.02	2.34
LE03619.1	1.49	1.67	1.60	1.62	4.15	2.91	1.66	1.70	1.16	0.00	1.76	2.43
LE11478.1	2.74	2.14	2.38	2.54	NaN	NaN	2.99	1.90	2.02	1.76	0.00	1.50
LE19854.1	2.88	2.23	2.19	3.03	NaN	NaN	2.10	1.87	2.34	2.43	1.50	0.00

Supplementary Fig. S3. An ML tree of the *L. erythrorhizon*'s AAT/SAT-like family, and its associated heat map of the Ks values (The Ks values are based on Supplementary Table S14).

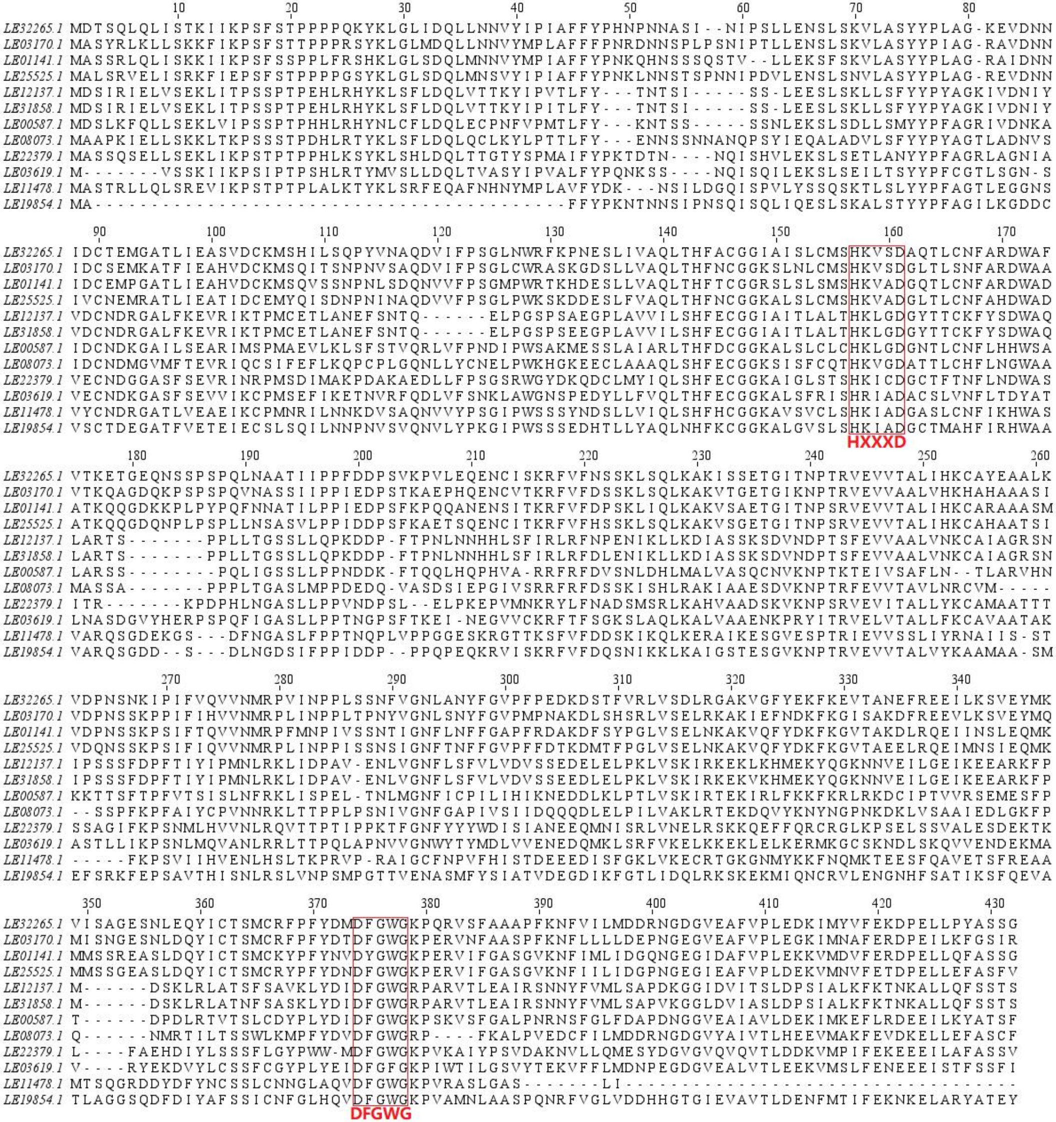

Supplementary Fig. S4. The complete AA alignment (after trimAl) of the *L. erythrorhizon*'s AAT/SAT-like family members (Red boxes: two conserved motifs 'HXXXD' and 'DFGWG').
